# Assessment of Hemoglobin Variants in Patients Receiving Health Care at the Ho Teaching Hospital: A Three-Year Retrospective Study

**DOI:** 10.1155/2020/7369731

**Published:** 2020-03-21

**Authors:** Daniel Kpodji Awaitey, Elliot Elikplim Akorsu, Emmanuel Allote Allotey, David Annor Kwasie, Precious Kwablah Kwadzokpui, Philip Apraku Tawiah, Stephen Adomako Amankwah, Albert Abaka-Yawson

**Affiliations:** ^1^Department of Medical Laboratory Sciences, School of Allied Health Sciences, University of Health and Allied Sciences, Ho, Ghana; ^2^Blood Bank, Laboratory Department, Ho Teaching Hospital, Ho, Ghana; ^3^Department of Pharmacognosy and Herbal Medicine, School of Pharmacy, University of Health and Allied Sciences, Ho, Ghana

## Abstract

**Background:**

It is estimated that one out of every three Ghanaians has hemoglobin genotype mutation. This change in genetic make-up may result in genotypes such as HbAS, HbSS, and HbSC. Many children in low- and middle-income countries die even before they are diagnosed with sickle cell disease (SCD). In Africa, there are limited data on the incidence and prevalence of SCD and the Volta region of Ghana is no exception.

**Aim:**

The aim of this study was to determine the prevalence of SCD and to assess the hemoglobin variants among patients attending Ho Teaching Hospital.

**Methods:**

A retrospective study design was used to extract information from the Hospital Administration and Management Systems (HAMS) on the hemoglobin electrophoresis results and corresponding full blood count results of the SCD and sickle cell anemia (SCA) patients as well as patients who were asked to do Hb electrophoresis irrespective of their sickling status. Data were collected for the period January 2016 to December 2018. Sickle cell disease status was determined using the Hb genotypes from the Hb electrophoresis results. The full blood count was used to categorize the severity of anemia based on the hemoglobin concentration in the SCA and SCD patients.

**Results:**

A total of 1,523 subjects were included in the study of which the prevalence for sickle cell disease was 16.7%. The SCD genotypes included HbS (6.2%), HbSC (7.9%), and HbSF (2.6%). Hemoglobin C disease (HbCC) constituted 0.3% out of the total prevalence of SCD. The prevalence of anemia was 99.2%, with the severest form in HbS. Also, majority of the SCD patients had severe anemia. Difference in the severity of anemia was found to be significant among both male (*P*=0.006) and female (*P*=0.004) participants with SCD.

**Conclusion:**

Patients receiving health care at the Ho Teaching Hospital had different hemoglobin variants with HbAS recording the highest prevalence. The high incidence of hemoglobin AS implies the possibility of having an increased population of individuals with sickle cell disease in future if measures are not put in place to improve screening, counseling, and education of the public about the health threat SCD poses.

## 1. Background

Over 1,000 human hemoglobin variants with single amino acid substitutions which lead to physiological implications with varying severity have been discovered [1]. HbS is the most common pathological hemoglobin mutation due to substitutional modification globally [2,3]. Other frequent and common variants of the hemoglobin are HbC, HbD, and HbE. In Africa, HbS is the most predominant pathological hemoglobin type whilst HbD and HbE are among Indian and Southeast Asian populations, respectively [4]. HbA2 fractions and fetal hemoglobin (HbF) can be increased in thalassemia, a disease which affects the synthesis of alpha- or beta-globin chains of the hemoglobin. Beta thalassemia can also occur in the presence of HbS and HbE. In the geographical area of the Mediterranean Sea, a combined sickle/beta thalassemia traits occur most frequently [4]. The sickle hemoglobin mutation is a structural variant of normal adult hemoglobin A which results from a single-point mutation where the amino acid glutamic acid is replaced with valine in position 6 of the *β*-globin chain of hemoglobin A [5]. Carriers or heterozygote (AS) individuals inherit an HbS allele from one parent and HbA allele from the other, but are, however, usually asymptomatic while homozygote (SS) individuals who inherit HbS alleles from both parents have the disease genotype and suffer from sickle cell anemia, which often leads to acute and chronic complications [6]. The homozygous (SS) sickle hemoglobin variant due to its hemolytic effect is referred to as sickle cell anemia (SCA), which largely is the most commonly inherited genetic disorder in the world and the most common form of the sickle cell disease. Persons with HbSS and HbS*β*° have the most severe forms of sickle cell disease (SCD) [7,8]. Without treatment, which is rarely available in low-income high-burden countries, it is assumed that most children born with the disease die in their first years of life [6]. Sickle cell disease is a genetic blood disorder affecting red blood cells, with a resulting high morbidity and mortality rates; the common forms of which are HbSS, HbSC, HbSD, HbSE, and HbS *β*°/*β* thalassemia [9,10]. Worldwide, out of the 330,000 babies born with major hemoglobinopathies, 275,000 have SCD, making it the major global hemoglobinopathy [2,11,12]. It is also estimated that 240,000 children are born with SCD yearly in sub-Saharan Africa [13]. This clearly supports reports of the United Nations General Assembly which recognized SCD as a global public health concern due to its high morbidity and mortality as well as the significant social and economic impact that results from the disease [14]. SCD patients in the developed world account for only 10% of the world's SCD patient population [11]. Life expectancy among individuals with the homozygous sickle hemoglobin mutation (HbSS) living in first-world countries, is estimated at a mean age of 39 years [15]. In the Volta region of Ghana, there is paucity of data on the prevalence of the condition among occupants of the region. This study, therefore, determined the prevalence of sickle cell disease among patients attending Ho Teaching Hospital and also assessed the hemoglobin variants presented by the participants.

## 2. Materials and Method

### 2.1. Study Design/Eligibility Criteria

A hospital-based retrospective study was used in this research to extract data on participants who visited the Ho Teaching Hospital between January 1, 2016 and December 31, 2018. All hematological reports with incomplete patient data, e.g., age, gender, and test results were excluded, whereas complete records within the stipulated period were reviewed and included in the study.

## 3. Data Collection

Data were collected from the Hospital Administrative and Management System (HAMS) of the Ho Teaching Hospital, Ho, Ghana. All hemoglobin electrophoresis results with full blood counts for patients who reported to the facility between January 2016 and December 2018 were reviewed. Prior to the data collection, the hemoglobin electrophoresis test in the laboratory during the period under review was found to be performed by using the DY-300 Electrophoresis Machine, Micromed, UK, using the alkaline cellulose acetate technique as described by Cheesbrough [16]. The parameters reviewed included sickling, hemoglobin (Hb) electrophoresis, and full blood count (FBC) test results of participants (SCA and other SCD patients). The total record of patients reviewed for the study was 1523.

## 4. Data Analysis

Data were entered and cleaned in Microsoft Excel spreadsheet and then imported into SPSS statistical software version 22.00 and GraphPad Prism statistical software version for statistical analysis. Normality of all continuous variables was tested. Continuous parametric variables were expressed as mean and standard deviation; continuous nonparametric variables were expressed as median (minimum and maximum) while categorical variables were expressed as frequency and percentages. Comparisons of parameters performed using unpaired *t* tests, one-way ANOVA, Kruskal–Wallis test, chi-square (*x*^2^) test, or Fisher exact test were appropriate. A *P* < 0.05 was considered as statistically significant for all analyses.

## 5. Ethical Issues

Ethical approval was obtained from the Ethical Review Committee of the University of Health and Allied Sciences. Approval to collect data from the HAMS was obtained from the facility's Ethical Committee on Human Research at the Ho Teaching Hospital (HTH). Information from the records of patients were kept confidential and used for the research purpose only.

## 6. Results

### 6.1. Demographic Characteristics of Study Participants

A total of 1523 participants were included in this study. Of these, 32.37% were males while 67.63% were females. Female population was generally higher in the years 2016 and 2017, except for 2018 where male proportion was larger. There was no significant gender difference observed in the years considered for this study (*P*=0.604). The greater proportion, 30.1%, of the study participants were between 25 and 34 years. In the years 2016, 2017, and 2018, majority of the participants were less than five years (36.71%), 35–60 years (39.90%), and greater than 61 years (43.75%), respectively. Age difference (0.013) in these years was statistically significant. The most predominant hemoglobin genotypes identified were HbAS, 40.32% (614/1523) and HbA, 37.29% (568/1523). Prevalence of hemoglobin SC, SF, and S were 7.9%, 2.6%, and 6.2%, respectively. Prevalence of sickle cell anemia HbSS was high in the year 2018 (51.6%) after a lower prevalence of 16.8% was recorded the year before (2017). The hemoglobin variants difference (0.005) in the years considered was statistically significant (Table 1).

### 6.2. Demographic Pattern of SCD among Study Participants

255 SCD cases were recorded in this study, majority of which were females (56.47%), unlike in the year 2016 (total of 111), where more males with SCD (36.94%) were recorded. Consecutively, higher proportions of females with SCD were recorded in the subsequent years 2017 (34.03%) and 2018 (38.19%). Gender differences in these years were statistically not significant (*P*=0.297). The greater proportion of SCD cases recorded generally was within 5–14 years of age. Majority of participants diagnosed of SCD in the year 2016 were younger (5–14 years) (35.37%) than those diagnosed in the subsequent years 2017, 50.0% (35–60 years) and 2018, 44.68% (15–24 years). There was no significant difference between the ages of the participants for the selected years (*P*=0.433). HbSS was the second most prevalent (37.3%), whereas SCD hemoglobin variant recorded next to hemoglobin SC, 47.45%. However, in 2016 and 2017, HbSF was the most prevalent SCD hemoglobin variant recorded (48.72% and 46.15%, respectively) while sickle cell anemia (HbSS) was the most prevalent in the year 2018 (51.58%). The SCD hemoglobin genotype difference in the years considered was statistically significant (*p* ≤ 0.001) (Table 2).

### 6.3. SCD Prevalence Pattern from 2016 to 2018

**Figure 1:**
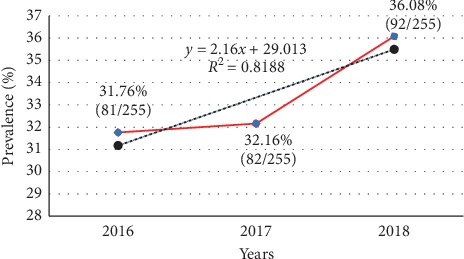
Annual prevalence pattern of SCD among the study participants.

Prevalence of SCD generally increased with advancement in years with 2018, recording a predominantly high prevalence of the disease (36.06%). Meanwhile, about 82% of the variations in the prevalence rate are accounted for by the variation in the years (*R*^2^ = 0.8188) (Figure 1).

### 6.4. Degrees of Anemia Stratified by the Various Sickle Cell Disease Genotypes among Males and Females

Degree of anemia was graded using the WHO grading of anemia (Hb ≥ 11 = normal; Hb: 9.5–10.9 = mild; Hb: 8–9.4 = moderate; Hb: 6.5–7.9 = severe). Two hundred and fifty-five of the participants were diagnosed of SCD. Of these, one hundred and thirteen representing 44.31% had their complete full blood count available at the time of the study. Out of this proportion, 55.75% (63/113) were females while 43.36% (59/113) were males. Majority of the male participants (46.94%) presented with HbSC while that of the female population presented with HbSS. Almost equal proportions of males (63.16%) and females (62.07%) who presented with HbSS were severely anemic. Generally, severe anemia was predominant between both sexes. Difference in the degrees of anemia presented by participants with SCD Hb genotype was significant between both male (*P*=0.006) and female (0.004) (Table 3).

### 6.5. Hematological Disparities among Participants Diagnosed with SCD

Generally, significantly elevated WBC counts were recorded across the various SCD Hb genotypes identified (SC, SS, and SF). However, only differential monocyte count was significantly high and predominant among participants with HbSC (53.05 ± 17.12; *P* < 0.001) genotype. Neutrophils, lymphocytes, eosinophils, and basophils elevations observed were not significantly different among the various SCD Hb genotypes. Significantly low Hb concentrations, RBC counts, and HCT were recorded across all three SCD hemoglobin genotypes but predominantly among participants with HbS and HbSF. Participants who had HbS presented with elevated PLT counts (Table 4).

## 7. Discussion

Hemoglobin variants are probably relatively common in Africa and several hundreds have been described [17]. Over the years, control and management of Hb disorders have encountered major economic and organizational difficulties [18]. Mutations in hemoglobin can cause a wide range of phenotypic outcomes, including anemia due to protein instability and red cell lysis [19] and occlusion of the microvascular circulation leading to tissue ischaemia, infarction, and chronic hemolytic anemia [20]. Knowledge in hemoglobin variants and carrier status is thus necessary in reproductive decision making to modify risk for offspring of serious conditions such as sickle cell anemia as well as provide direct health benefit to carriers [21]. This study sought to evaluate hemoglobin variants in patients who visited the Ho Teaching Hospital, Ghana. A total of 1523 patients' data were retrieved for the three-year period under review (2016–2018). The prevalence of HbAS, HbA, HbSC, HbS, HbAC, HbSF, HbC, and HbAF among the study population during the three-year period was estimated at 40.3%, 37.3%, 7.9%, 6.2%, 5.2%, 2.6%, and 0.3%, respectively. Contrary to findings in this study, in South Western Nigeria, HbAS was the second most frequent hemoglobin genotype with a frequency distribution of 24.5% next to HbA with a frequency distribution of 65.3%, whereas HBAC and HbSC recorded 4.0% and 1.1%, respectively [22]. Similarly, in the Niger Delta of Nigeria, 69.1% subjects had HbA, 29.4% had the sickle cell trait (HbAS), and 1.5% had sickle cell anemia (HbS) [23]. The high prevalence of HbAS recorded in this study implies that in the absence of health policy regulations comprising intensive health education, the possibility of having an increased population of sickle cell disease individuals in the proximate future is very high as inheritance of one copy of an abnormal hemoglobin variant (C, S, and F) in addition to an S hemoglobin makes an individual diseased [24]. Sickle cell disease patients experience enormous clinical complications as a result of changes in their hematological parameters vis-à-vis development of cerebrovascular disease and cognitive impairment as well as recurrent episodes of vasoocclusion and inflammation often resulting in progressive damage to most organs, such as the brain, kidneys, lungs, bones, and cardiovascular system [8]. This study recorded two hundred and fifty-five SCD cases (overall prevalence of 16.7%), majority of which were females 56.47% (144/255) who recorded the highest proportion in the years 2017, 34.03% (49/114) and 2018, 38.19% (55/114). Majority of participants diagnosed of SCD in the year 2016, 35.37% (29/82) were younger (5–14 years) than those diagnosed in the subsequent years 2017, 50.0% (35–60 years) and 2018, 44.68% (15–24 years).

Additionally, findings from this study indicated that the prevalence of SCD generally increased from 2016 to 2018 with 2018 recording a high prevalence of the disease (36.06%), 2017 (32.16%), and 2016 (31.76%) indicating that without intensified education and counseling of most, especially carriers of the condition, the current proportion may continue to upsurge. In this study, the hematological profile of sickle cell disease patients reviewed within the period of study (2016–2018) was assessed. Significantly low Hb concentrations, RBC counts, and HCT was recorded across all three SCD hemoglobin genotypes but predominantly among participants with HbS and HbSF which largely could be due to the crenation and subsequent lysing outcome of the RBCs. A comparable outcome was recorded among akin participants in North Maharashtra, India, where low levels of Hb concentration, RBC count, and PCV was documented [25]. Likewise, Patel and colleagues [26] also reported significant low level of hemoglobin and HCT among patients with HbS genotype. The mean cell volume recorded in this study was high, supporting the known fact that MCV usually is high in SCD patients due to the increasing need of erythropoiesis in response to chronic hemolysis leading to macrocytosis [27]. Compared to other studies which documented low MCH levels [27], this study recorded significantly low mean corpuscular hemoglobin levels. Except for participants with HbS, this study recorded normal PLT counts among the study participants. Elevated PLT counts recorded could be attributed to the outcome of the interaction between the sickled cells and the endothelial cells as a result of the vasoocclusion during microcirculation [8]. Generally, patients with HbS are known to have a significantly higher mean total WBC count [28] and findings from this study were not an exemption. Out of the 113 participants whose hematological parameters were retrieved, majority of both males and females with HbSS were severely anemic, as reported by Jadhav [29].

## 8. Conclusion

This study revealed that various hemoglobin variants are present in patients receiving health care at the Ho Teaching Hospital, Ghana. The high prevalence of HbAS genotype recorded implies that the possibility of having an increased population of sickle cell disease individuals in the next few years is high.

## 9. Recommendations

Education on sickle cell disease must be intensified in order to curb the high prevalence of the condition.

## Figures and Tables

**Table 1:** Participants' demographic details stratified by year of testing.

Parameters	Total	Year category			*P* value
		2016	2017	2018	
Gender	1523 (100.00)	430 (28.23)	507 (33.29)	586 (38.48)	
Male	493 (32.37)	138 (27.99)	157 (31.85)	198 (40.16)	0.604
Female	1030 (67.63)	292 (28.35)	350 (33.98)	388 (37.67)	

*Age group*					
<5	237 (15.56)	87 (36.71)	67 (28.27)	83 (35.02)	**0.013**
5–14	258 (16.94)	80 (31.01)	90 (34.88)	88 (34.11)	
15–24	344 (22.59)	84 (24.42)	115 (33.43)	145 (42.15)	
25–34	459 (30.14)	122 (26.58)	151 (32.90)	186 (40.52)	
35–60	193 (12.67)	46 (23.83)	77 (39.90)	70 (36.27)	
≥61	32 (2.10)	11 (34.38)	7 (21.88)	14 (43.75)	

*Hb variants*					
AA	568 (37.29)	157 (27.64)	194 (34.15)	217 (38.20)	**0.005**
AC	79 (5.19)	17 (21.52)	25 (31.65)	37 (46.84)	
AS	614 (40.32)	175 (28.50)	203 (33.06)	236 (38.44)	
AF	3 (0.20)	0 (0.00)	2 (66.67)	1 (33.33)	
CC	4 (0.26)	0 (0.00)	1 (25.00)	3 (75.00)	
SC	121 (7.94)	32 (26.45)	48 (39.67)	41 (33.88)	
SF	39 (2.56)	19 (48.72)	18 (46.15)	2 (5.13)	
SS	95 (6.24)	30 (31.58)	16 (16.84)	49 (51.58)	

Data are presented as frequency with corresponding percentages in parenthesis. *P* value is significant at <0.05.

**Table 2:** Annual SCD pattern among the study participants.

Parameters	Total	Year category			*P* value
		2016	2017	2018	
	255 (100.00)	81 (31.76)	82 (32.16)	92 (36.08)	
*Gender*
Male	111 (43.53)	41 (36.94)	33 (29.73)	37 (33.33)	0.297
Female	144 (56.47)	40 (27.78)	49 (34.03)	55 (38.19)	

*Age group*					
<5	64 (25.10)	22 (34.38)	24 (37.50)	18 (28.13)	0.433
5–14	82 (32.16)	29 (35.37)	26 (31.71)	27 (32.93)	
15–24	47 (18.43)	12 (25.53)	14 (29.79)	21 (44.68)	
25–34	43 (16.86)	15 (34.88)	9 (20.93)	19 (44.19)	
35–60	16 (6.27)	2 (12.50)	8 (50.00)	6 (37.50)	
≥61	3 (1.18)	1 (33.33)	1 (33.33)	1 (33.33)	

*Hb variants*					
SC	121 (47.45)	32 (26.45)	48 (39.67)	41 (33.88)	<0.001
SF	39 (15.29)	19 (48.72)	18 (46.15)	2 (5.13)	
SS	95 (37.25)	30 (31.58)	16 (16.84)	49 (51.58)	

**Table 3:** Gender-based classification anemia stratified by Hb genotype.

	Total	Grade of anemia				*P* value
		Severe	Moderate	Mild	Normal	
*Males*						
Hb genotype	49 (43.36)	19 (38.78)	10 (20.41)	11 (22.45)	9 (18.37)	
SC	23 (46.94)	2 (8.70)	6 (26.09)	8 (34.78)	7 (30.43)	0.006
SS	19 (38.78)	12 (63.16)	3 (15.79)	3 (15.79)	1 (5.26)	
SF	7 (14.29)	5 (71.43)	1 (14.29)	0 (0.00)	1 (14.29)	

*Females*						
Hb genotype	63 (55.75)	26 (41.27)	17 (26.98)	12 (19.05)	8 (12.70)	
SC	24 (38.10)	3 (12.50)	7 (29.17)	9 (37.50)	5 (20.83)	0.004
SS	29 (46.03)	18 (62.07)	7 (24.14)	1 (3.45)	3 (10.34)	
SF	10 (15.87)	5 (50.00)	3 (30.00)	2 (20.00)	0 (0.00)	

Data are presented as frequency with corresponding percentage in parenthesis. *P* value is significant at <0.05.

**Table 4:** Hematological parameters stratified by SCD genotypes.

FBC parameters	SCD genotypes			*P* value
	SC	S	SF	
WBC	9.95 (4.72–24.00)	15.95 (4.32–79.63)	16.61 (7.82–81.80)	<0.001
RBC	4.04 ± 0.77	2.94 ± 0.92	2.57 ± 1.03	<0.001
Hb	10.10 (3.90–14.90)	7.55 (3.30–13.10)	7.20 (2.00–11.50)	<0.001
HCT	27.60 (4.70–39.30)	22.40 (9.20–36.10)	19.40 (5.80–31.20)	<0.001
MCV	69.60 (54.50–108.00)	78.90 (53.60–121.00)	81.70 (63.90–137.00)	<0.001
MCH	24.80 (19.70–36.00)	27.05 (20.00–38.50)	28.20 (22.40–40.80)	<0.001
MCHC	37.00 (29.20–40.10)	34.70 (29.90–38.00)	26.00 (29.80–39.70)	0.010
PLT	247.60 ± 128.10	308.70 ± 144.60	271.60 ± 114.70	**0.091**
NUET%	53.05 ± 17.12	51.49 ± 15.43	47.07 ± 15.15	**0.427**
LYMP%	36.05 ± 14.38	37.57 ± 15.62	40.45 ± 14.60	**0.578**
MONO%	53.05 ± 17.12	8.43 ± 3.50	8.79 ± 2.74	<0.001
EO%	1.30 (0.10–7.10)	1.85 (0.10–17.50)	0.80 (0.10–13.60)	**0.480**
BASO%	0.40 (0.10–5.10)	0.40 (0.10–24.10)	0.40 (0.10–30.60)	**0.552**
RDW-SD	40.90 (29.80–60.00)	54.05 (34.00–90.60)	55.60 (40.40–68.10)	<0.001
RDW-CV	16.20 (11.10–24.20)	20.00 (11.10–100.00)	18.40 (13.80–90.00)	<0.001

Data are presented as mean ± standard deviation for continuous parametric variables and median (minimum–maximum) for continuous nonparametric variables. Comparisons of parameters were performed using one-way ANOVA for parametric variables and Kruskal–Wallis test for nonparametric variables. A *P* < 0.05 was considered statistically significant for all analyses.

## Data Availability

The data used to support the study are available upon request from the corresponding author.
